# Polydatin Attenuates Cisplatin-Induced Acute Kidney Injury by Inhibiting Ferroptosis

**DOI:** 10.1155/2022/9947191

**Published:** 2022-01-15

**Authors:** Lu Zhou, Peng Yu, Ting-ting Wang, Yi-wei Du, Yang Chen, Zhen Li, Man-lin He, Lan Feng, Hui-rong Li, Xiao Han, Heng Ma, Hong-bao Liu

**Affiliations:** ^1^Department of Nephrology, Tangdu Hospital, Fourth Military Medical University, Xi'an 710038, China; ^2^Shaanxi University of Chinese Medicine, Xianyang 712046, China; ^3^Department of Physiology and Pathophysiology, School of Basic Medical Sciences, Fourth Military Medical University, Xi'an 710032, China

## Abstract

Cisplatin is widely used in the treatment of solid tumors, but its application is greatly limited due to its nephrotoxicity; thus, there is still no effective medicine for the treatment of cisplatin-induced acute kidney injury (Cis-AKI). We previously identified that polydatin (PD) exerts nephroprotective effects by antioxidative stress in AKI models. Recent evidence suggests that oxidative stress-induced molecular events overlap with the process of ferroptosis and that there are common molecular targets, such as glutathione (GSH) depletion and lipid peroxidation. Nevertheless, whether the nephroprotective effect of PD is related to anti-ferroptosis remains unclear. In this study, the inhibitory effect of PD on ferroptosis was observed in both cisplatin-treated HK-2 cells (20 *μ*M) in vitro and a Cis-AKI mouse model (20 mg/kg, intraperitoneally) in vivo, characterized by the reversion of excessive intracellular free iron accumulation and reactive oxygen species (ROS) generation, a decrease in malondialdehyde (MDA) content and GSH depletion, and an increase in glutathione peroxidase-4 (GPx4) activity. Remarkably, PD dose-dependently alleviated cell death induced by the system Xc^−^ inhibitor erastin (10 *μ*M), and the effect of the 40 *μ*M dose of PD was more obvious than that of ferrostatin-1 (1 *μ*M) and deferoxamine (DFO, 100 *μ*M), classical ferroptosis inhibitors. Our results provide insight into nephroprotection with PD in Cis-AKI by inhibiting ferroptosis via maintenance of the system Xc^−^-GSH-GPx4 axis and iron metabolism.

## 1. Introduction

Cisplatin is a chemotherapeutic agent widely used to treat various malignancies, and its application is greatly limited due to its nephrotoxicity, including its onset of acute kidney injury (AKI) [1]. Undoubtedly, effective drugs to prevent cisplatin-induced AKI (Cis-AKI) in clinical practice are in urgent demand. Therefore, it is very important to further investigate the pathophysiology of Cis-AKI and its effective therapeutic drugs.

Several studies have shown that DNA damage, oxidative stress, inflammation, vascular dysfunction, and mitochondrial damage may be involved in the pathogenesis of Cis-AKI [1]. Recently, several studies [2–6] have suggested that cisplatin treatment leads to excessive lipid peroxidation, ferritinophagy-mediated free iron release, and a decrease in the activity of glutathione peroxidase-4 (GPx4), indicating the close link between ferroptosis and Cis-AKI. Hence, ferroptosis intervention may be an effective strategy to attenuate Cis-AKI; nevertheless, there have not been specific drugs against ferroptosis in clinical application to date.

Polydatin (PD, C_20_H_22_O_8_) is a natural active ingredient extracted from the dried roots of the perennial herb *Polygonum cuspidatum* Sieb. et Zucc., which might play a potential therapeutic role in various kidney disorders, such as AKI [7–16], diabetic nephropathy [17–24], lupus nephritis [25], and hyperuricemia [26–28], and has been used to treat patients with irritable bowel syndrome in Europe [29]; moreover, PD has been shown to exhibit mechanisms to antioxidant stress, anti-inflammation, and antifibrosis characteristics; improvement of mitochondrial damage; and regulation of autophagy [9, 30, 31]. Resveratrol, an aglycone of PD, has been verified to ameliorate myocardial and liver damage caused by iron overload [32–34]; moreover, it has been confirmed that resveratrol can reduce iron load in hemodialysis patients in clinical studies [35]. Recent studies have noted that PD [36] or resveratrol [37, 38] can inhibit ferroptosis to ameliorate myocardial ischemia-reperfusion injury and brain injury. However, the role of PD in Cis-AKI is still unclear. Our previous studies have demonstrated that PD, which is capable of increasing the levels of glutathione (GSH) and GPx and reducing the content of malondialdehyde (MDA), plays a renal protective role in the renal ischemia-reperfusion injury model through antioxidative stress and anti-inflammatory mechanisms [13–15]. A series of molecular events induced by oxidative stress overlap with ferroptosis processes after AKI, with common molecular targets such as lipid peroxidation and GSH depletion. All of the evidence above strongly indicates that PD may be a latent therapeutic compound against ferroptosis in AKI.

Considering that the antiferroptotic effect of PD in AKI has not yet been clearly reported, the purpose of this study was to investigate the relationship between the nephroprotective effect and antiferroptotic role in Cis-AKI to provide solid evidence for the research and development of nephroprotective agents for clinical AKI treatment.

## 2. Materials and Methods

### 2.1. Chemicals and Reagents

Cisplatin, dimethyl sulfoxide (DMSO), Hoechst 33342 fluorescent staining kit (B2261), and dihydroethidium (DHE) fluorescent staining kit (D7008) were purchased from Sigma-Aldrich (St. Louis, MO, USA). Polydatin (PD, MB5448) and ferrostatin-1 (Fer-1, MB4718) were acquired from Meilun Biotech (Dalian, China). Deferoxamine (DFO, HY-B0988) was purchased from MedChemexpress LLC (Princeton, USA). Lipid peroxidation malondialdehyde (MDA) assay kit (S0131M), reactive oxygen species (ROS) assay kit (S0033M), DCFH-DA fluorescent probe assay (S0033S), mitochondrial membrane potential assay kit with JC-1 (C2006), and cellular glutathione peroxidase (GPX) assay kit with NADPH (S0056) were purchased from Beyotime Biotechnology (Shanghai, China). CheKine™ Reduced Glutathione (GSH) Colorimetric Assay Kit (KTB1600) was purchased from Abbkine (Wuhan, China). 4 Hydroxynonenal (4HNE) ELISA assay kit was purchased from Yanqi Biotech (Shanghai, China). Anti-4HNE antibody (ab46545) and lipid hydroperoxide (LPO) assay kit (ab133085) was acquired from Abcam (ON, Canada). Phosphate-buffered saline (PBS), Dulbecco's modified Eagle's medium/nutrient mixture F-12 (DMEM/F-12), fetal bovine serum (FBS), Hanks' balanced salt solution (HBSS), cell counting kit (CCK-8), DAPI fluorescence staining kit (G1012), and fluorescein (FITC) Tunel cell apoptosis detection kit (TUNEL, G1501) were purchased from Servicebio Technology (Wuhan, China). FeRhoNox-1 fluorescent probe (MX4558) was purchased from Maokang Biotech (Shanghai, China). HK-2(GDC0152) was purchased from China Center for Type Culture Collection (Wuhan, China).

### 2.2. Animals and Experimental Protocol

All animal experiments were conducted in strict accordance with the Guidelines of Health and guidelines for use and were permitted by the Scientific Investigation Committee of the Fourth Military Medical University. Male C57BL/6 mice (8-10 weeks of age, weight 20-25 g) were purchased from Experimental Animal Center of the Fourth Military Medical University (Xi'an, China) and bred in an experimental animal room of SPF grade. They were randomly divided into four groups: control (equivalent saline containing 1% DMSO) group (*n* = 5), cisplatin (20 mg/kg dissolved in saline) only group (*n* = 7), cisplatin + polydatin (40 mg/kg dissolved in 1% DMSO) group (*n* = 7), and cisplatin+ Fer-1 (5 mg/kg dissolved in 1% DMSO) group (*n* = 7) were administered intraperitoneally. Mice were injected with cisplatin once; PD or Fer-1 was given 1 h before and 24 h after cisplatin. Animals were ethically sacrificed by dislocating their spines at 48 h after cisplatin injection, and whole blood and kidneys were collected for further analysis.

### 2.3. Blood Physiochemical Assays

The whole blood drawn from the retroocular vein plexus was centrifuged at 4°C, 4000 rpm, for 10 min to acquire the serum sample. The level of blood urea nitrogen (BUN) and serum creatinine (Scr) was measured according to manufacturer's instructions using the urea determination kit (C013-2-1, Nanjing Jiancheng, China) and creatinine determination kit (C011-2-1, Nanjing Jiancheng, China), respectively.

### 2.4. Renal Tissue Histopathological, Immunohistochemistry (IHC), and Terminal Deoxynucleotidyl Transferase dUTP Nick-End Labeling (TUNEL) Assay

Fresh renal tissues were washed with ice-cold stroke-physiological saline solution, fixed overnight with 10% neutral buffered formalin, and then paraffin-embedded, followed cut into a thickness of 4 *μ*m sections, which were used for hematoxylin-eosin (H&E) staining, or TUNEL fluorescent staining, or 4HNE immunohistochemistry according to manufacturer's instructions. Evaluation of histological score of kidney injuries (HSK) was performed by a renal pathologist under the blinded manner. The kidney sections representing a minimum of 100 fields of at least 5 mice from each group were semiquantitatively assessed. HSK was scored using a 4-point quantitative scale, as previously described by us [13, 14]: 0 represented normal histology; 1 represented mild injuries [nuclear lost (necrosis) less than 1/3 tubular section]; 2 represented moderate damage [more than 1/3 and less than 2/3 of a tubular cross section shows nuclear loss (necrosis)]; 3 represented the severe damage [more than 2/3 of nuclear loss (necrosis) per tubular cross-section]. We calculated the total score for each kidney slice and added all 10 scores to a maximum possible injury score of 30.

### 2.5. Mitochondrial Morphology Observation by Transmission Electron Microscopy (TEM)

Simply, 1 mm^3^ of the fresh renal cortex was removed and quickly placed in a TEM fixative at 4°C. Tissues were embedded and cut into ultrathin sections at 60–80 nm, and then, uranium lead double staining was performed. The ultrastructure of the kidney was observed by TEM, and the images were collected.

### 2.6. Determination of Lipid Peroxidesand Lipid Peroxidation

The lipid peroxide (LPO) and MDA levels in supernatants from both the renal tissues and cultured HK-2 cells were measured using a commercially available kit, and absorbance was measured at 500 nm and 535 nm using a spectrophotometer, respectively, according to manufacturer's instructions [16]. In addition, HK-2 cells were also incubated with C11-BODIPY581/591 (D3861, Invitrogen, USA) and DAPI in flow cytometry buffer (2% FCS, 2 mM EDTA in PBS) at 37°C for 10 to 30 min in darkness. The cells were subsequently washed with PBS and resuspended in FACS buffer for flow cytometry (excitation 480 nm, emission 510 nm and 590 nm) [39]. BODIPY-positive cells among DAPI-negative cells were analyzed as compared to a control sample using BODIPY measurement.

### 2.7. Determination of GSH Levels and GPx Activity

The GSH levels and GPx4 activity in supernatants from both the renal tissues and cultured HK-2 cells were determined using CheKine™ Reduced GSH Colorimetric Assay Kit and Cellular Glutathione Peroxidase Assay Kit with NADPH, respectively, according to manufacturer's instructions [5]. The absorbance of each sample was measured at 412 nm for GSH and at 340 nm for GPx4 using a spectrophotometer. The GSH concentration and GPx4 activity were calculated according to the standard curve. A total of three independent repeats were performed.

### 2.8. Cell Culture and Treatment

Human proximal tubular epithelial cells (HK-2 cells) were obtained from the China Center for Type Culture Collection (GDC0152, Wuhan, China), grown in DMEM/F-12 supplemented with 10% heat-inactivated FBS, 100 U/ml penicillin, and 100 *μ*g/ml streptomycin in a humidified atmosphere of 5% CO_2_ and 95%air at 37°C. Exponentially growing HK-2 cells were seeded at 2 to 4 × 10^5^ cells/well in six-well culture plates and cultured for 1 day before each experiment. Then, the cells were divided into four groups: control group, cisplatin (20 *μ*M) group, cisplatin + PD (40 *μ*M) group, and cisplatin + Fer-1 (1 *μ*M) group.

### 2.9. Cell Viability Assay

HK-2 cells were cultured without or with different concentrations of PD (10, 20, and 40 *μ*M), DFO (100 *μ*M) or Fer-1 (1 *μ*M) in presence or absence of cisplatin (20 *μ*M), and the cell viability was measured by CCK-8 according to manufacturer's instructions. So, as to evaluate the effect of PD on ferroptosis, in some experiments, we replaced cisplatin with erastin (10 *μ*M).

### 2.10. Determination of Intracellular Labile Iron

The cellular labile iron pool (LIP) is a compartment of nonferritin-bound iron that can generate oxygen radicals via the Fenton reaction resulting in oxidative stress and cell injury, including ferroptosis. Intracellular LIP in the kidney tissue was measured by electron paramagnetic resonance (EPR) spectroscopy (Magnettech GmbH, Germany) as described in Woodmansee and Imlay [40] with modifications. Mice kidney samples were homogenized in 10 mM Tris–HCl buffer, 120 mM KCl (pH 7.4), and 1 mM DFO. Samples were incubated on ice for 1 h and then rapidly frozen with liquid nitrogen in 4 mm O.D. EPR tubes. Under the condition of central magnetic field 1600 G, sweep width 800 G, microwave frequency 9.75 GHz, microwave power 20 mW, modulation frequency 50 kHz, modulation amplitude 4.759 G, and time constant 81.92 ms, EPR spectra were recorded. All samples were analyzed three times; the sample was removed from the EPR cavity each time and then repositioned within the cavity prior to initiating spectral scanning.

The content of labile iron in HK-2 cells was monitored using selective Fe^2+^ fluorescent probe FeRhoNox-1, calcein-chelatable cytosolic LIP assay, and Iron Assay Kit (Sigma-Aldrich, MAK025). FeRhoNox-1, which is a turn-on fluorescent probe specific for the detection of labile iron Fe^2+^, was used to detect intracellular LIP, and the cellular distribution of FeRhoNox-1 was consistent with Golgi [41]. HK-2 cells were grown to confluence in 35 mm laser confocal petri dishes in DMEM, and PD (40 *μ*M) or Fer-1 (1 *μ*M) was added in the absence or presence of cisplatin (20 *μ*M). Cells were incubated with 5 *μ*M FeRhoNox-1 for 1 h prior to assays. Cells were washed twice with PBS before staining nuclei with Hoechst 33342. The fluorescence was immediately observed with a confocal laser-scanning microscope (CLSM, ECLIPSE Ti, Nikon, Tokyo, Japan).

Calcein acetoxymethyl ester (Calcein-AM, Corning Inc., Corning, NY, USA), which is the most widely adopted labile iron fluorescent probe, was used to detect cytosolic LIP. Calcein-AM is a nonfluorescent lipophilic ester that passes through the cellular membrane and reacts with cytosolic unspecific esterases to produce calcein, a fluorochromic alcohol that chelates labile iron under quenching of the green fluorescence [42]. Briefly, the cells were loaded at 37°C with 2 *μ*M calcein for 30 min and then washed with HBSS. DFO was added at a final concentration of 100 *μ*M to remove iron from calcein, leading to dequenching. Fluorescence changes with the addition of DFO were used for indirect measurement of the LIP. Fluorescence was measured at 485 nm excitation and 535 nm emissions with a fluorescence plate reader.

In addition, intracellular level of ferrous (Fe^2+^) iron was also determined using an iron assay kit from Sigma-Aldrich (MAK025), according to manufacturer's instructions. Absorbance was measured at 593 nm using a microplate reader.

### 2.11. Mitochondrial Membrane Potential (MMP) by Florescent JC-1

The MMP was measured using JC-1 fluorescent probes, a monomer present in the cytosol (staining green), which also aggregated in the normally polarized mitochondria (which stains red). However, in damaged and depolarized mitochondria, JC-1 exists as a monomer and stains the cytosol green. The change of fluorescence emission from red to green indicates mitochondrial depolarization. HK-2 cells were incubated with 5 *μ*mol/l JC-1 for 15 min at 37°C. A minimum of 104 cells per sample was analyzed using a FACS Calibur flow cytometer (Becton Dickinson, BD Biosciences, USA). Data were analyzed using BD FACSuite software. The percentage of cells with abnormally low MMP (i.e., green fluorescence) was also measured.

### 2.12. ROS Detection

Dihydroethidium (DHE) fluorescence was used to detect the ROS levels in the renal tissues. The renal tissues were immersed in saccharose (30% *w*/*v*), embedded at the optimal cutting temperature (OCT), and stored at -20°C until immunofluorescence assay. The OCT blocks were cut into10 *μ*m in a cryostat and mounted on polylysine-coated glass slides. Tissue sections were incubated in 10 *μ*M DHE for 30 min at 37°C in a humidified chamber protected from light, then incubated with DAPI solution at room temperature for 10 min, kept in a dark place. In the presence of superoxide anion, DHE is oxidized to ethidium, which produces a bright red fluorescence. After washing with PBS, sections were mounted and visualized by CLSM.

The intracellular ROS levels of cells were monitored using the DCFH-DA fluorescent probe assay. After entering the cells, DCFH-DA is hydrolyzed by esterases to 2′,7′-dichlorofluorescin (DCFH), which is captured in the cell. In the presence of ROS, the nonfluorescent DCFH is oxidized and turns into highly fluorescent 2′,7′-dichlorofluorescein (DCF). HK-2 cells were seeded on 35 mm laser confocal petri dishes at a density of 1.0 × 10^6^ cells per well and cultured at 37°C for 24 h with 5% CO_2_. The cells were treated with PD (40 *μ*M) or Fer-1 (1 *μ*M) for 24 h in the absence or presence of cisplatin (20 *μ*M). The cell culture medium was replaced with DCFH-DA. After incubation for 20 min in dark at 37°C, the cells were rinsed using PBS three times to remove the extracellular DCFH-DA. Hoechst 33342 (blue) was added at 37°C for 5 min following manufacturer's instruction. All procedures were done in the dark, and the samples were observed by CLSM.

### 2.13. Statistical Analysis

Data were presented as the mean ± standard deviation (SD). Differences between the data means were compared by use of Student's *t* test and one-way analysis of variance (ANOVA) followed by Dunnett's multiple comparison tests using the SPSS statistical software package (SPSS, Inc., Chicago, IL, USA), and *P* value of less than 0.05 was considered to be statistically significant.

## 3. Results

### 3.1. Effects of PD on Attenuating Renal Injury in Cis-AKI Mice

**Figure 1:**
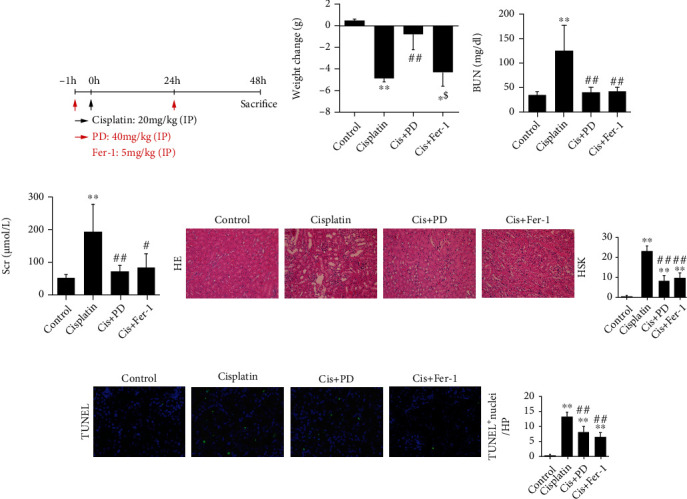
Therapeutical effects of PD on attenuating renal injury in Cis-AKI mice. (a) Mice pretreated with PD (40 mg/kg) or Fer-1 (5 mg/kg) were administered with intraperitoneal injections of cisplatin (20 mg/kg), and either PD or Fer-1 was intraperitoneally reinjected at 24 h after cisplatin injection. They were executed 48 h after the cisplatin injection. (b–d) Body weight changes, BUN, and Scr were measured at 48 h after injection of cisplatin. (e, f) Histopathology analysis of the kidneys in Cis-AKI mice was performed by hematoxylin-eosin (H&E) staining (400x magnification), and the HSK was graded. (g) Representative terminal deoxynucleotidyl transferase dUTP nick-end labeling- (TUNEL-) stained sections of the kidney (200x magnification). ∗*P* < 0.05, ∗∗*P* < 0.01 vs. control; ^#^*P* < 0.05, ^##^*P* < 0.01 vs. cisplatin; ^$^*P* < 0.05, ^$$^*P* < 0.01 vs. Cis+PD (*n* = 5 to 7). Cis: cisplatin; PD: polydatin; Fer-1: ferrostatin-1; BUN: blood urea nitrogen; Scr: serum creatinine; HSK: histological score of kidney injuries.

We initially evaluated the role of PD in Cis-AKI and compared it with ferrostatin-1 (Fer-1), a specific ferroptosis inhibitor. To this end, PD (40 mg/kg) or Fer-1 (5 mg/kg) was intraperitoneally injected 1 h before cisplatin injection and then reinjected 24 h after cisplatin injection. The Cis-AKI mice were killed 48 h after cisplatin injection (Figure 1(a)).

Compared with that of the control group, the body weight of Cis-AKI mice was significantly decreased, whereas the body weight of Cis-AKI mice pretreated with PD was obviously improved (Figure 1(b)). Cisplatin treatment resulted in an increase in serum biochemical parameters such as blood urea nitrogen (BUN, Figure 1(c)) and serum creatinine (Scr, Figure 1(d)). Both, PD and Fer-1 treatment, showed suppression in cisplatin-induced injury (Figures 1(c) and 1(d)). Histological examinations were also assessed at 48 h after cisplatin treatment. As expected, compared with control kidneys from vehicle-treated mice, cisplatin increased the histological score of kidney (HSK) value (Figure 1(e)) and the number of apoptotic cells in the TUNEL assay (Figure 1(f)). Conversely, pretreatment with PD or Fer-1 significantly reduced HSK and TUNEL-positive cells (Figures 1(e) and 1(f)).

### 3.2. Effects of PD on Inhibiting Ferroptosis in Cis-AKI Mice

**Figure 2:**
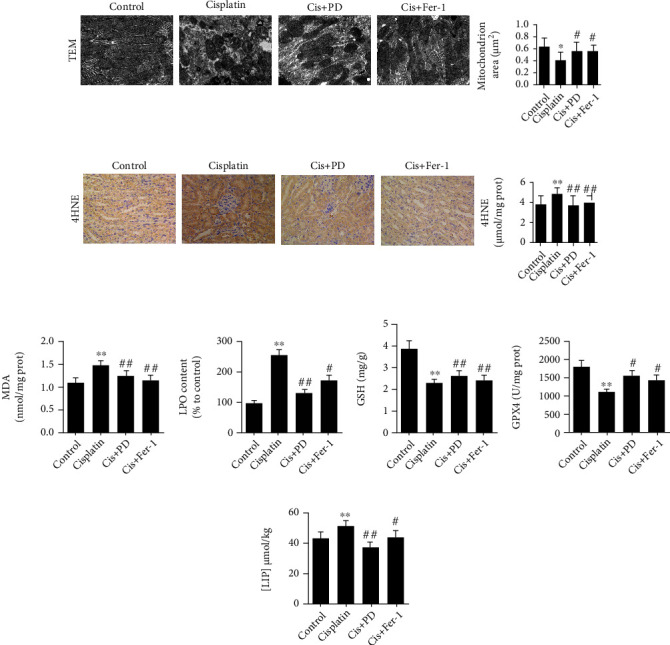
Effects of PD on inhibiting ferroptosis in Cis-AKI mice. (a) The ultrastructure of the renal tissue was seized by TEM at 48 h after cisplatin injection (12000x magnification). (b–d) The lipid peroxidation markers, immunohistochemistry for 4HNE (400x magnification), and determination of MDA and LPO were performed at 48 h after cisplatin injection. (e, f) The level of GSH and the activity of GPx4 were tested in kidneys at 48 h after cisplatin injection. (g) Levels of chelatable iron in kidneys were determined by EPR detection of ferrioxamine. ∗*P* < 0.05, ∗∗*P* < 0.01 vs. control; ^#^*P* < 0.05, ^##^*P* < 0.01 vs. cisplatin; ^$^*P* < 0.05, ^$$^*P* < 0.01 vs. Cis+PD (*n* = 5 to 7). Cis: cisplatin; PD: polydatin; Fer-1: ferrostatin-1; TEM: transmission electron microscopy; 4HNE: 4-hydroxynonenal; MDA: malondialdehyde; LPO: lipid hydroperoxide; GSH: glutathione; GPx4: glutathione peroxidase-4; LIP: labile iron pool.

We found that PD have nephroprotective effects similar to Fer-1, however, that much higher doses of PD were used, which prompted us to further explore the impact of PD on ferroptosis in Cis-AKI mice. For this purpose, ultrastructural shifts and lipid peroxidation levels (a key feature of ferroptosis) were first determined. TEM observation showed no significant changes in the mitochondrial structure of the renal tissue in vehicle-treated mice (Figure 2(a)). In Cis-AKI mice, ferroptosis-associated mitochondrial changes, such as increased membrane density, reduced mitochondrial volume, and decreased or absent mitochondrial cristae, were observed in renal tissue (Figure 2(a)), which were significantly alleviated to a similar degree by pretreatment with 40 mg/kg PD or 5 mg/kg Fer-1 (Figure 2(a)).

Lipid peroxidation is the key driver of ferroptosis. Detection of the lipid peroxidation markers 4-hydroxynonenal (4HNE, Figure 2(b)), MDA (Figure 2(c)), and lipid peroxides (LPOs, Figure 2(d)) revealed that cisplatin could induce the increase of 4HNE, MDA, and LPOs levels in the kidneys, and the levels of 4HNE, MDA, and LPOs were significantly decreased by PD or Fer-1 pretreatment (Figures 2(b)–2(d)), indicating that PD is able to reverse the aberrant lipid peroxidation occurring during Cis-AKI.

Depletion of GSH or inhibition of the GSH-dependent antioxidant enzyme GPx4 is also a critical feature of ferroptosis. Inspired by the above data, we also examined the level of GSH and the activity of GPx4. The data showed that the GSH level (Figure 2(e)) and GPx4 activity (Figure 2(f)) decreased 48 h after Cis-AKI, which could be rescued by PD or Fer-1 pretreatment (Figures 2(e) and 2(f)).

Ferroptosis depends on excess accumulation of free iron, which is an essential component of lipid peroxidation. Therefore, electron paramagnetic resonance (EPR) was used to detect the labile iron pool (LIP) in mouse kidney homogenates in this study. As expected, LIP in kidney homogenates was increased by cisplatin treatment, and PD or Fer-1 pretreatment mitigated the cisplatin-induced increase in LIP (Figure 2(g)).

### 3.3. Inhibition of PD on Cisplatin-Induced Ferroptosis in HK-2 Cells

Since we observed the protective effect of PD against ferroptosis in Cis-AKI mice in vivo, we further verified the effect of PD on erastin (10 *μ*M, a specific inducer of ferroptosis)-induced cell toxicity in HK-2 cells in vitro.

**Figure 3:**
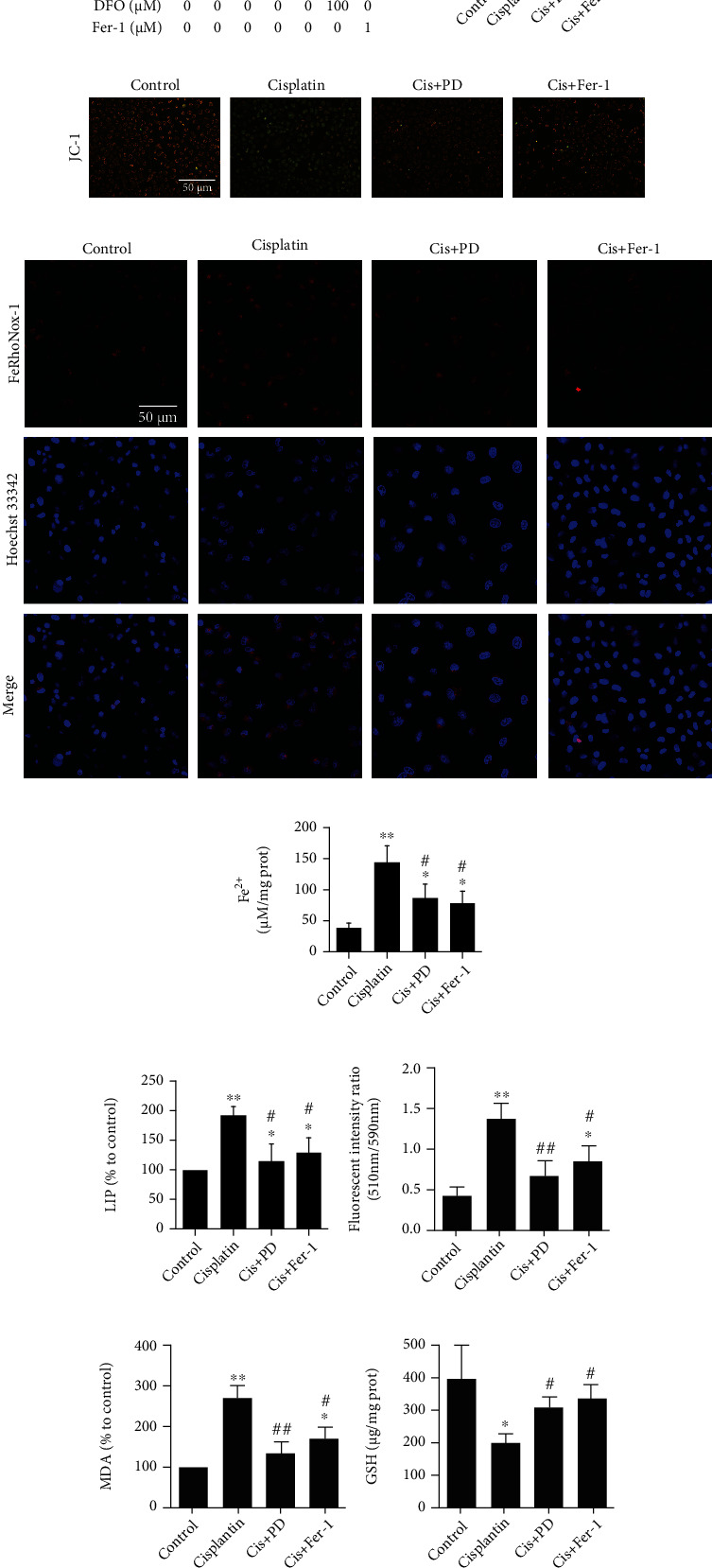
Inhibition of PD on cisplatin-induced ferroptosis in HK-2 cells. (a) CCK8 showing the viability of HK-2 cells under the treatment of different concentration of PD (10, 20, and 40 *μ*M), DFO (100 *μ*M), or Fer-1 (1 *μ*M) in presence or absence of erastin (20 *μ*M). (b) CCK8 showing the viability of HK-2 cells under the simultaneous treatment of different concentration of cisplatin for 24 h. (c) CCK8 showing the viability of HK-2 cells under the treatment of different concentration of PD (10, 20, and 40 *μ*M), DFO (100 *μ*M), or Fer-1 (1 *μ*M) with or without cisplatin (20 *μ*M). (d, e) Changes in MMP expression levels were detected by fluorescence microscope and flow cytometry using JC-1. (f–h) Intracellular labile iron in HK-2 cells was measured using selective Fe (II) fluorescent probe FeRhoNox-1, iron assay kit, and LIP assay. (i, j) The lipid peroxidation in HK-2 cells was measured using C11-BODIPY581/591 and MDA assay. (k, l) The level of GSH and the GPx4 activity of HK-2 cells were examined in 24 h after cisplatin injection. ∗*P* < 0.05, ∗∗*P* < 0.01 vs. control (nondrug treated); ^#^*P* < 0.05, ^##^*P* < 0.01 vs. cisplatin (or erastin) alone; ^$^*P* < 0.05, ^$$^*P* < 0.01 vs. Cis (or erastin)+40 *μ*M PD (*n* = 6). Cis: cisplatin; PD: polydatin; Fer-1: ferrostatin-1; DFO: deferoxamine; LIP: labile iron pool; MDA: malondialdehyde; GSH: glutathione; GPx4: glutathione peroxidase-4.

The results from the cell counting kit-8 (CCK-8) assay showed that treatment with erastin for 30 h significantly inhibited cell viability, and treatment with PD dose-dependently alleviated erastin-induced cell death in HK-2 cells compared with nondrug-treated HK-2 cells. The effect of the 40 *μ*M dose of PD was more obvious than that of Fer-1 (1 *μ*M) and deferoxamine (DFO) (100 *μ*M) (Figure 3(a)).

The toxicity of cisplatin on HK-2 cells was detected by CCK-8 assay, and the protective effect of PD was compared with that of Fer-1 (1 *μ*M) and DFO (100 *μ*M). Cisplatin (20 *μ*M) exposure for 24 h significantly inhibited the viability of HK-2 cells compared with the control group (Figure 3(b)). Therefore, a dose of 20 *μ*M cisplatin was used in the subsequent experiments. The protective effect of PD on cell viability after cisplatin treatment was dose-dependent, with 40 *μ*M as the optimal concentration (Figure 3(c)). Based on this result, 40 *μ*M PD was used for the subsequent experiments.

We also analyzed mitochondrial membrane potential (MMP) by florescent JC-1 in cisplatin-induced damage of HK-2 cells treated with or without PD or Fer-1, and the results showed that both PD and Fer-1 could reverse the mitochondrial damage induced by cisplatin (Figures 3(d) and 3(e)).

The intracellular labile iron and ferrous iron (Fe^2+^) levels were detected using the FeRhoNox™-1 fluorescent probe (Figures 3(f)), Fe^2+^ assay (Figures 3(g)), and calcein AM-chelatable LIP assay (Figure 3(h)). The results indicated a marked increase in intracellular labile iron and Fe^2+^ levels in HK-2 cells after cisplatin treatment, which was attenuated in PD-treated or Fer-1-treated cells.

Lipid peroxidation was confirmed by C11 BODIPY 581/591 staining (Figure 3(i)) and MDA assay (Figure 3(j)), and the results show that the lipid peroxidation in HK-2 cells was increased by cisplatin treatment, which could be rescued by 40 *μ*M dose of PD treatment, and the effect was even stronger than that of 1 *μ*M Fer-1.

The measurement of GSH level (Figure 3(k)) and GPx4 activity (Figure 3(l)) showed that both PD and Fer-1 treatment significantly mitigated the decrease in GSH level and GPx4 activity after cisplatin induction.

### 3.4. PD Limits Intracellular ROS Increase Induced by Cisplatin In Vitro and In Vivo

**Figure 4:**
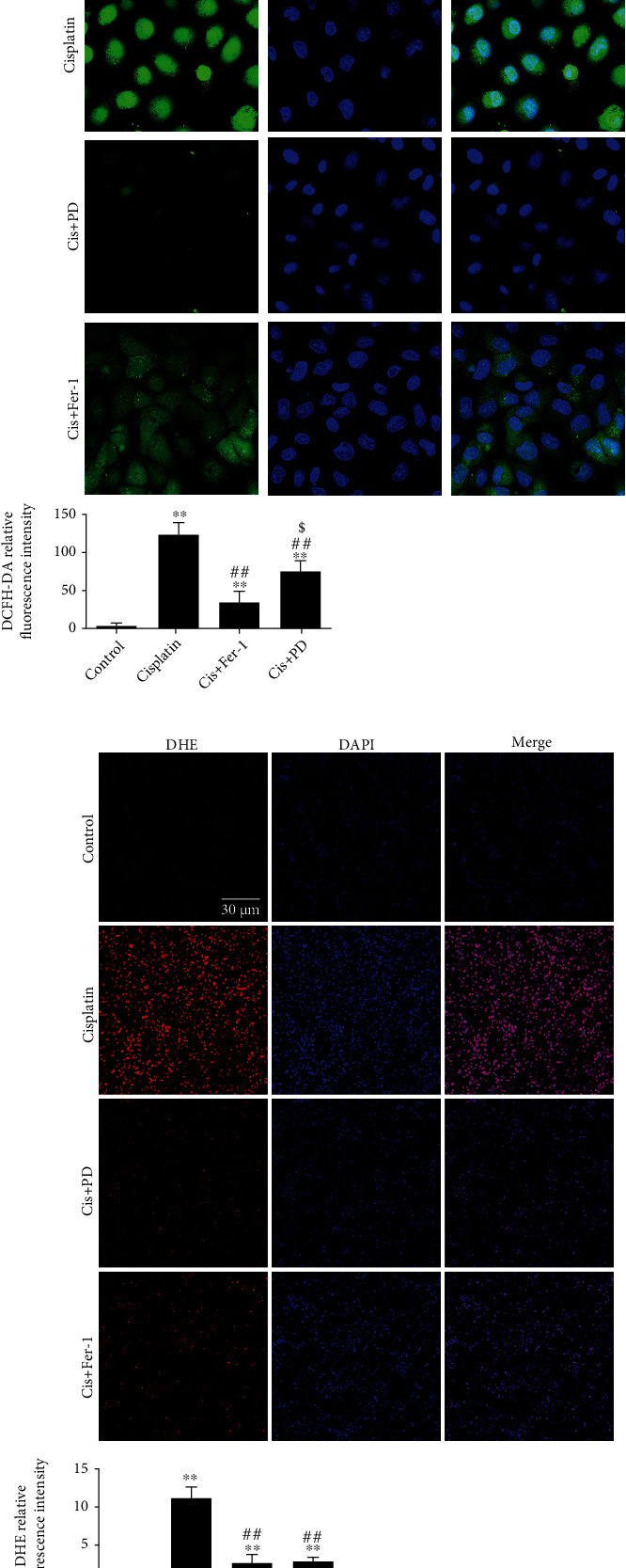
PD limits intracellular ROS increase induced by cisplatin in vitro and in vivo. (a) The ROS level of HK-2 cells was captured by CLSM at 24 h after cisplatin injection (DCFH-DA fluorescent staining, 600x magnification). (b) The ROS level of kidney tissues was captured by CLSM at 48 h after cisplatin injection (DHE fluorescent staining, 200x magnification). ∗*P* < 0.05, ∗∗*P* < 0.01 vs. control; ^#^*P* < 0.05, ^##^*P* < 0.01 vs. cisplatin; ^$^*P* < 0.05, ^$$^*P* < 0.01 vs. Cis+PD (*n* = 5–7). Cis: cisplatin; PD: polydatin; Fer-1: ferrostatin-1; DCFH-DA: 2′,7′-dichlorofluorescin diacetate; DHE: dihydroethidium.

Excessive Fe^2+^ levels in cells induce a large amount of reactive oxygen free radicals, which further attack and oxidize cell membrane lipids by triggering ferroptosis. In this study, intracellular reactive oxygen species (ROS) were measured by using DCFH-DA fluorescent probes for cisplatin-induced HK-2 cells and DHE fluorescent probes for the kidneys of Cis-AKI mice. Compared with that of the control group, the ROS levels of HK-2 cells exposed to cisplatin significantly increased, which decreased after supplementation with a 40 *μ*M dose of PD, and the effect was even stronger than that of a 1 *μ*M dose of Fer-1 (Figure 4(a)).

Likewise, the level of intracellular ROS in kidneys measured by the fluorescent probe DHE was increased in Cis-AKI mice compared with that of the control and was ameliorated in PD (40 mg/kg)-pretreated Cis-AKI mice, and its effect was almost comparable to that of Fer-1(5 mg/kg) (Figure 4(b)).

## 4. Discussion

Recommended as a first-line chemotherapeutic, cisplatin is applied in the treatment of various types of malignancies, including lymphoma, germ cell carcinomas, and neoplasms of the prostate, bladder, and lung [1]. Cisplatin freely passes through glomerular filter and is largely reabsorbed by proximal tubules (especially S3 segment tubules), and such localized accumulation may result in nephrotoxicity [43]. As early as 30 years ago, researchers proposed that cisplatin-induced free iron overload leads to lipid peroxidation in kidneys, but the link between ferroptosis and Cis-AKI has only recently been recognized. Denget al. [5] confirmed that cisplatin induced free iron release, GSH depletion, a decrease in GPx4 activity and excessive lipid peroxidation in vivo and in vitro, which was subsequently verified by other groups of investigators [2–4]. Ferroptosis, officially named in 2012, is characterized by intracellular iron accumulation and lipid peroxidation apart from classical apoptosis, necrosis, and autophagy [44]. Activating or inhibiting ferroptosis has become a new strategy for treating various diseases [45]. Exploring more potential nephroprotective compounds for ferroptosis will provide an experimental basis for their clinical treatment. In this study, we investigated the nephroprotective effect of PD and elucidated its possible antiferroptotic mechanism. Our innovative findings include defining abnormal activation of ferroptosis in Cis-AKI and demonstrating that PD contributes to protection against Cis-AKI by inhibiting ferroptosis through reducing excessive free iron accumulation and increasing GPx4 activity.

Studies using the ferroptosis inducer erastin have shown that the system Xc^−^-GSH-GPx4 axis plays a central role in limiting lipid peroxidation and ferroptosis [46]. Lipid peroxidation due to GSH depletion is a key feature of ferroptosis. GPx4 is a lipid enzyme that can catalyze the conversion of GSH to glutathione disulfide (GSSG) in the oxidation reaction and then remove excess peroxides, subsequently alleviating the peroxidation of polyunsaturated fatty acids in the membrane [47]. Cisplatin has a high affinity for mercaptan-rich biomolecules, including GSH. In the cytoplasm, the majority of intracellular cisplatin is conjugated to GSH to form the Pt-GS complex [48]. Similar to erastin, depletion of GSH along with the inactivation of GPxs was the underlying mechanism of action for cisplatin. In recent years, several studies [49–55] found the nephroprotective effects of PD and its aglycone and resveratrol, against cisplatin-induced oxidative stress and inflammation. However, their effects on inhibiting ferroptosis have only recently been reported in myocardial ischemia-reperfusion injury and intracerebral hemorrhage models [36–38]. Our results identified that a 40 mg/kg dose of PD significantly rescued the depletion of GSH and the decrease in GPx4 activity after cisplatin induction, and its effect was almost equivalent to that of a 5 mg/kg dose of Fer-1. Erastin, a potent and selective inhibitor of system Xc^−^ [56], can lead to GSH depletion and loss of GPx4 activity by preventing cystine import and then reduce the clearance of lipid peroxide and induce ferroptosis. In this study, we determined that PD dose-dependently alleviated erastin-induced cell death in HK-2 cells, and 40 *μ*M PD has a significant inhibitory effect on ferroptosis. These results suggest that PD may exert a regulatory effect on the system Xc^−^-GSH-GPx4 axis, which may be an important antiferroptotic mechanism of PD.

Ferroptosis is dependent on excessive accumulation of free iron (Fe^2+^), which is a crucial component of lipid oxidation [45]. Fe^2+^ is partially transferred by ferritin and can be partly stored in the LIP. Abnormal iron homeostasis and excess intracellular Fe^2+^ levels lead to the production of a quantity of reactive oxygen free radicals, which further attack and oxidize cell membrane lipids, resulting in ferroptosis [45]. Resveratrol, an aglycone of PD, has been widely demonstrated to ameliorate organ damage caused by iron overload. A recent study noted that PD can dramatically reduce the deposition of iron in traumatic brain injury [36], although its mechanism was not discussed in detail. Iron chelators, including DFO, prevent free radical production and delay ferroptosis. In previous studies, polyphenols were often found to be potent iron chelators, and several polyphenol compounds have been reported to chelate iron and regulate iron homeostasis, such as proanthocyanidin, curcumin, quercetin, and silymarin [57–60]. In the present study, we verified that PD could significantly inhibit excessive intracellular Fe^2+^ accumulation and ROS generation both in the kidneys of Cis-AKI mice in vivo and in HK-2 cells after cisplatin induction in vitro. PD, as a natural polyphenolic compound, has strong biological activity and remains to have unique potential to regulate iron metabolism, although its specific mechanism remains to be further clarified. This study confirmed that PD inhibits ferroptosis in cisplatin-induced AKI in vitro and in vivo, although PD requires higher dose than the traditional ferroptosis inhibitor Fer-1. PD has comprehensive effects, such as antioxidative, anti-inflammation, and autophagy-regulating effects, in addition to its inactivation of ferroptosis, as well as its superior potential for clinical application. However, we still propose that further exploration of the effect and mechanism of PD in AKI and chronic kidney disease will provide a new therapeutic option for all nephrology practitioners. Furthermore, it is not clear whether the action of PD might be referable to iron chelation, free radical scavenging, or other actions on iron handling.

## 5. Conclusions

In conclusion, *in vitro* and *in vivo* experiments indicated the prominent nephroprotective effects of PD against ferroptosis in Cis-AKI models, occurred at least partly through inhibiting excessive intracellular free iron accumulation and ROS production, rescuing GSH consumption, and enhancing GPx4 activity, thereby decreasing lipid peroxidation and ferroptosis sensitivity and ultimately attenuating the pathological progression of AKI. Although the specific molecular mechanism of PD against ferroptosis has still not been comprehensively determined, the current work might provide novel insight into the potential clinical application of PD as a drug for AKI treatment.

## Data Availability

All data related to this paper may also be requested from the corresponding authors (email: xjsnlhb@fmmu.edu.cn).

## Abbreviations

AKI: Acute kidney injury
Cis-AKI: Cisplatin-induced AKI
GPx4: Glutathione peroxidase-4
PD: Polydatin
GSH: Glutathione
MDA: Malondialdehyde
DHE: Dihydroethidium
Fer-1: Ferrostatin-1
DFO: Deferoxamine
ROS: Reactive oxygen species
4HNE: 4-Hydroxynonenal
LPO: Lipid hydroperoxide
BUN: Blood urea nitrogen
Scr: Serum creatinine
TUNEL: Terminal deoxynucleotidyl transferase dUTP nick-end labeling
HSK: Histological score of kidney injuries
TEM: Transmission electron microscopy
EPR: Electron paramagnetic resonance
LIP: Labile iron pool
CLSM: Confocal laser-scanning microscope
MMP: Mitochondrial membrane potential.
